# Stromal vascular fraction-enriched fat grafting as treatment of adherent scars: study design of a non-randomized early phase trial

**DOI:** 10.1186/s13063-022-06514-3

**Published:** 2022-07-19

**Authors:** Linda Vriend, Joris A. van Dongen, Anouk Pijpe, Marianne K. Nieuwenhuis, Sandra J. M. Jongen, Martin C. Harmsen, Paul P. M. van Zuijlen, Berend van der Lei

**Affiliations:** 1 Department of Pathology & Medical Biology, University of Groningen, University Medical Center Groningen, Groningen, the Netherlands; 2 Department of Plastic Surgery, University of Groningen and University Medical Center of Groningen, Groningen, the Netherlands; 3 Department of Plastic Surgery, University of Utrecht, and University Medical Center Utrecht, Utrecht, the Netherlands; 4 Burn Center, Red Cross Hospital, Beverwijk, the Netherlands; 5 Association of Dutch Burn Centers, Red Cross Hospital, Beverwijk, the Netherlands; 6 Department of Plastic, Reconstructive and Hand Surgery, Amsterdam UMC, Vrije Universiteit Amsterdam, Amsterdam Movement Sciences, Amsterdam, the Netherlands; 7 Association of Dutch Burn Centers, Martini Hospital, Groningen, the Netherlands; 8 Research Group Healthy Ageing, Allied Health Care and Nursing, Hanze University of Applied Sciences, Groningen, the Netherlands; 9 Department of Human Movement Sciences, University Medical Center Groningen, Groningen, the Netherlands; 10 Pediatric Surgical Centre, Emma Children’s Hospital, Amsterdam UMC, University of Amsterdam, Vrije Universiteit, Amsterdam, the Netherlands; 11 Bergman Clinics, Rijswijk, the Netherlands; 12 Bergman Clinics, Heerenveen, the Netherlands

**Keywords:** Adherent scars, Scar quality, Burn scars, Stromal vascular fraction, Adipose-derived stromal cells, ASC, Fat grafting, Pliability

## Abstract

**Background:**

In the last decades, autologous fat grafting has been used to treat adherent dermal scars. The observed regenerative and scar-reducing properties have been mainly ascribed to the tissue-derived stromal vascular fraction (tSVF) in adipose tissue. Adipose tissue’s components augment local angiogenesis and mitosis in resident tissue cells. Moreover, it promotes collagen remodeling. We hypothesize that tSVF potentiates fat grafting-based treatment of adherent scars. Therefore, this study aims to investigate the effect of tSVF-enriched fat grafting on scar pliability over a 12-month period.

**Methods and design:**

A clinical multicenter non-randomized early phase trial will be conducted in two dedicated Dutch Burn Centers (Red Cross Hospital, Beverwijk, and Martini Hospital, Groningen). After informed consent, 46 patients (≥18 years) with adherent scars caused by burns, necrotic fasciitis, or degloving injury who have an indication for fat grafting will receive a sub-cicatricic tSVF-enriched fat graft. The primary outcome is the change in scar pliability measured by the Cutometer between pre- and 12 months post-grafting. Secondary outcomes are scar pliability (after 3 months), scar erythema, and melanin measured by the DSM II Colormeter; scar quality assessed by the patient and observer scales of the Patient and Observer Scar Assessment Scale (POSAS) 2.0; and histological analysis of scar biopsies (voluntary) and tSVF quality and composition. This study has been approved by the Dutch Central Committee for Clinical Research (CCMO), NL72094.000.20.

**Conclusion:**

This study will test the clinical efficacy of tSVF-enriched fat grafting to treat dermal scars while the underlying working mechanism will be probed into too.

**Trial registration:**

Dutch Trial Register NL 8461. Registered on 16 March 2020

## Background

The first fat grafting procedure was described in 1893 to treat a soft tissue defect for reconstructive purposes [1]. Nowadays, fat grafting is routinely used for both reconstructive as well as esthetic purposes and is far “more than volume correction alone” [2, 3]. In 2001, Zuk et al. described the adipocyte stromal cells (ASCs) that are present in large amounts in the stromal vascular fraction (SVF) of adipose tissue and possess multipotent capabilities [4]. This discovery led to an increased use of fat grafting for regenerative and scar-reducing purposes and to treat damaged tissues, such as burn wounds [5]. Since then, it is thought that observed beneficial effects are for a large part attributed to the SVF in adipose tissue.

SVF is easily obtained by dissociating lipoaspirate enzymatically or mechanically [6]. SVF comprises the non-adipocyte fraction of fat and is composed of stromal cells such as fibroblasts and other mesenchymal stromal cells as well as vasculature cells (endothelial and smooth muscle). The stromal cells produce and maintain the extracellular matrix that embeds and supports the cells in SVF. Recently, a reliable, fast, intraoperative mechanical isolation procedure has been developed to yield tissue-derived stromal vascular fraction (tSVF), which makes clinical application of tSVF more feasible than enzymatic isolated SVF (cellular SVF) [7–9]. On the one hand, tSVF probably effectuates better retention of paracrine factors through extracellular matrix components that lack in single cell suspensions like cSVF, where cells disappear through leakage of lymph vessels. On the other hand, tSVF is a unit of transplanted tissue that provides a nutritious environment, serving as a therapeutic component of adipose tissue. Case reports and retrospective studies have reported improved esthetic outcomes and pain relieve after autologous fat grafting of problematic and adherent scars [3, 5, 10–23]. These results hold promise but, besides the obvious methodological drawbacks of the study design, lack adequate scar evaluation with validated objective or subjective scar measurement tools. Moreover, physical, physiological, and psychological evaluation and measurements are also important since adherent scars cause physical burden, e.g., stiffness and impaired range of motion, and psychological burden, e.g., psychosocial distress [24, 25]. Several validated scar scales, e.g., the Patient and Observer Scar Assessment Scale (POSAS), Vancouver Scar Scale (VSS), and Manchester Scar Scale (MSS), have been developed to assess the aforementioned aspects of scar quality [26–28]. The POSAS is recognized as a highly reliable scar rating scale; moreover, it also includes the patients’ opinion and is therefore favorable for subjective scar assessment ([29], www.posas.org).

Recently, autologous fat grafting was effectively used to improve scar quality, in particular scar pliability and pain in adherent scars [30]. Scar pliability and color were assessed with validated objective measures, i.e., the Cutometer and DSM II Colormeter, and scar quality and pain with validated subjective measures, i.e., the POSAS patient and observer scales ([29, 31–37], www.posas.org). The results of the beforenamed studies warrant further research with SVF or ASCs, because these components are deemed responsible for a large part of the observed ameliorating effects on scar features [3, 5, 10–23, 30]. Moreover, enrichment of fat grafts with SVF may induce even larger and longer lasting ameliorating effects on scar features than plain autologous fat.

A well-designed clinical trial investigating these potential effects of SVF-enriched fat grafting is warranted. The aim of this study is to assess the efficacy of tSVF-enriched fat grafting in patients with adherent scars using a comprehensive scar evaluation protocol. This study will also provide benchmark data on histological change of adherent scars after tSVF-enriched fat grafting.

## Methods, design, and outcome measures

### Objectives

The aim of this study is to assess the efficacy of tSVF-enriched fat grafting in patients with adherent scars. The primary outcome measure is the change in scar pliability as assessed with the Cutometer Skin Elasticity Meter Dual MPA 580 (Courage and Khazaka GmbH, Cologne, Germany) pre- and 12 months post-tSVF-enriched grafting. The secondary outcome measures are scar erythema and melanin index, as a proxy for color and pigmentation, measured by the DSM II Colormeter (Cortex Technology, Hadsund, Denmark); scar quality assessed by the patient and observer scales of the Patient and Observer Scar Assessment Scale (POSAS 2.0); and histological features of tSVF and scar biopsies.

### Protocol and registration

This study was approved by the Dutch Central Committee for Clinical Research (CCMO) NL72094.000.20 and by the Institutional Review Boards of the participating hospitals (Red Cross Hospital, Beverwijk, and Martini Hospital, Groningen, the Netherlands). Methods of the study are specified in a protocol that is registered at the Dutch trial register (March 16, 2020, NL 8461) (https://www.trialregister.nl/trial/8461).

### Trial design

**Fig. 1:**
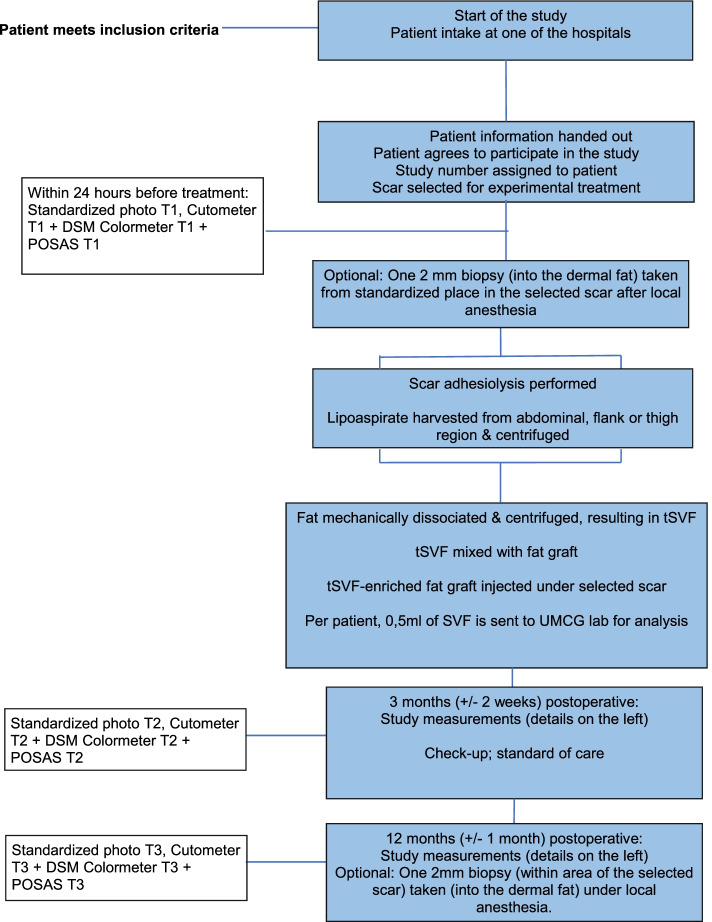
Prospective cohort study design

The trial design is a non-randomized early phase intra-patient, before-after trial on the effect of tSVF-enriched fat grafting on scar pliability in patients with adherent scars and who are treated in the Red Cross Hospital, Beverwijk, or the Martini Hospital, Groningen, the Netherlands (Fig. 1).

Other study designs were considered but were not feasible for several reasons. For example, splitting a scar in two areas (one side treated with SVF-enriched fat grafting, one with plain fat grafting as the control group) may potentially lead to shifting of treatments from one side to the other, leading to unreliable results. Moreover, the effect of tSVF-enriched fat grafting or plain fat grafting may not be limited to its own area; thus, the border between both may display a gradient effect. Treating two separate scars within one patient was also considered but would exclude patients that do not have two scars and scars may possibly be not comparable. Therefore, the non-randomized study design was chosen optimum to obtain reliable results.

### Participants

Patients, aged ≥18 years with adherent scars (≥12 months old) caused by burns, necrotic fasciitis, or degloving injury, visiting the outpatient clinic of one of the Burn Centers in Beverwijk or Groningen who have an indication for fat grafting are eligible for this trial. Exclusion criteria are previous scar treatment with fat grafting, skin melanoma in patients’ history, pregnant or lactating, BMI <18 (general exclusion criteria for fat grafting procedure), unlikely to comply with the requirements of the study protocol and follow-up, and insufficient knowledge of the Dutch language (Table 1). Patients are included after they received a confirmed understandable and neutral explanation of the study by a member of the research team and after signing informed consent following the guidelines of the CCMO.

**Table 1:** Inclusion and exclusion criteria

Inclusion criteria	Exclusion criteria
- Age ≥18 years - Patient has an adherent scar (minimum scar age: 12 months) caused by burns, necrotic fasciitis, or degloving injury, for which fat grafting is indicated - Competent adults - Patients seen by a plastic surgeon in Burn centers of the Red Cross Hospital, Beverwijk or Martini Hospital, Groningen, The Netherlands	- Previous scar treatment with fat grafting in selected scar - General exclusion criteria for fat grafting procedure: pregnancy, lactating, BMI < 18 kg/m^2^ - Skin melanoma in patient’s history - Unwillingness to commit to the study protocol and show up for all follow-up moments - Insufficient proficiency in Dutch to the extent that clear communication is not possible

### Scar selection

**Fig. 2:**
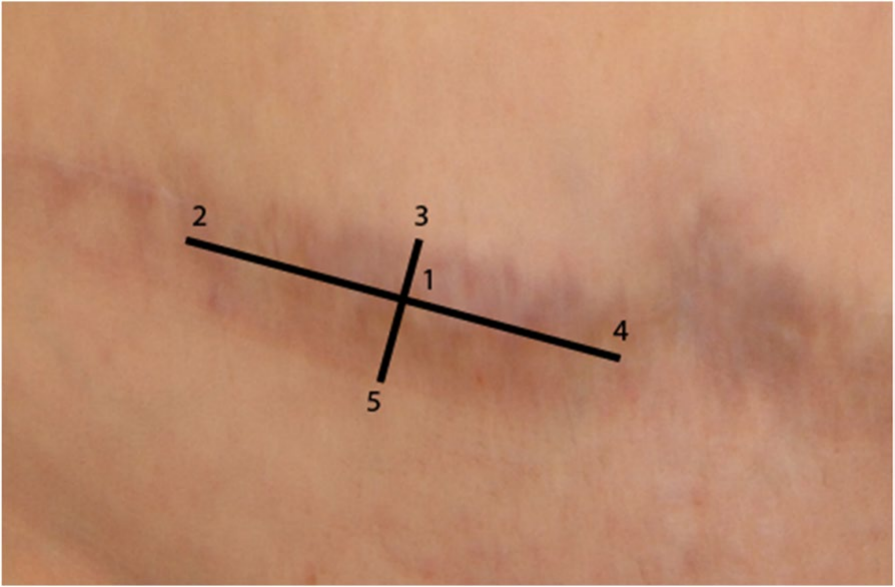
Selected scar divided in 5 points according to a standardized algorithm

Before surgery, one scar area will be selected and marked according to a standardized algorithm for objective data collection (Fig. 2) [30]. The maximum size of the scar area is limited by the maximum quantity of tSVF that can be obtained and a minimum length of 2 cm will apply. Each scar consists of five measurement points. The five points in the selected area will be subjected to all study outcomes.

### Surgical procedure

**Fig. 3:**
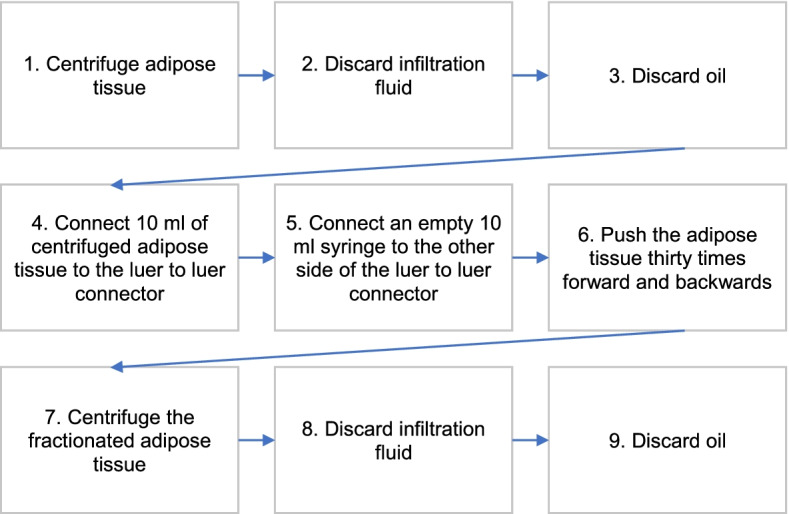
Flow diagram of the fractionation of adipose tissue procedure (FAT procedure)

**Fig. 4:**
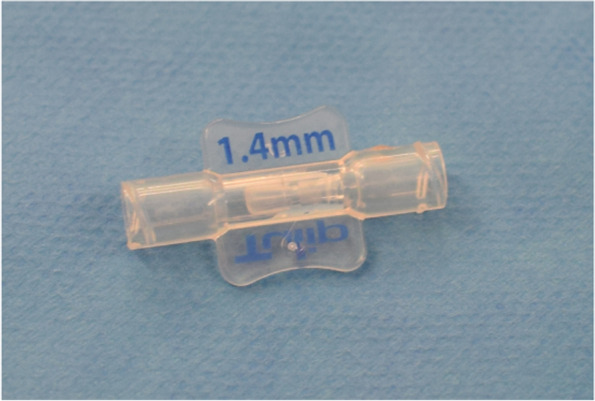
Frontal and side views of disposable fractionator with one hole of 1.4 mm inside, used to fractionate adipose tissue

**Fig. 5:**
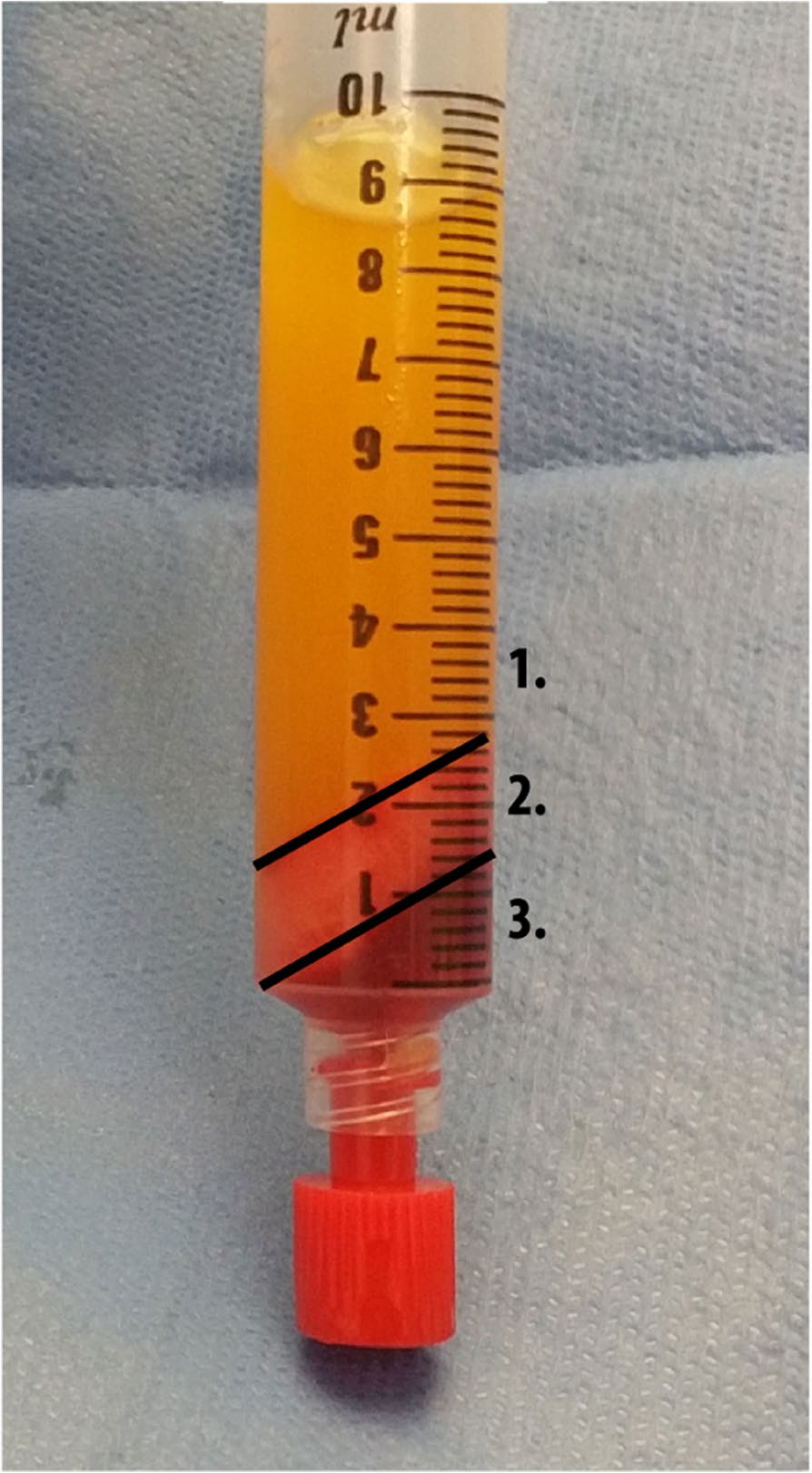
Lipoaspirate after performing the fractionation of adipose tissue procedure: (1) oil = disrupted adipocytes, (2) tissue stromal vascular fraction, and (3) infiltration fluid including pellet consisting of dead cell remainders

Plastic surgeons, who have had uniform training in fat grafting procedures and are also additionally trained in Dutch burn care centers, will carry out the study procedures. The results of this study will be directly translation to real-world setting, because in clinics the procedures will be executed by a similar set of plastic surgeons with similar fat grafting skills. Under general anesthesia, lipoaspirate is harvested from the abdominal wall, flank, or thigh with fine harvesting cannulas. Out of 10 ml lipoaspirate, 1 ml of tSVF will be produced intraoperatively with the fractionation of adipose tissue (FAT) procedure, a fat dissociation procedure using a fractionator (Figs. 3 and 4) [38]. For preparing the tSVF-enriched fat graft, 10 ml lipoaspirate in a 10-ml Luer-Lock syringe will be centrifuged at 3000 rpm with a 9.5-cm radius fixed angle rotor for 2.5 min (Medilite, Thermo Fisher Scientific, Waltham, MA) at room temperature (Fig. 5). Thereafter, the upper oily layer will be drained from the top while the bottom layer will be drained by removing the lower cap of the syringe. Subsequently, in the syringe, 9.0 ml of fat graft will be left to which is added 1.0 ml of tSVF: two syringes, one with 9 ml of fat and one with 1 ml of tSVF, will be connected to a Luer-Lock and the contents will be mixed by gently pushing the content of one syringe into the other, this way yielding the tSVF-enriched fat. Small incisions at the border of the scar are created to perform adhesiolysis of the selected scar. Then, the tSVF-enriched fat (tenfold excess fat over tSVF) will be injected under the scar. This procedure is repeated until the entire scar area has reached fat grafting saturation (when enriched fat graft starts coming out of the incision holes). After fat saturation, the scar is covered and fixed with gausses. Practically, 50 ml of fat has to be harvested to yield 25 ml of fat and 2.5 ml tSVF, resulting in 27.5 ml tSVF-enriched fat graft.

## Study outcomes

### Primary outcome measure

#### Scar pliability

**Fig. 6:**
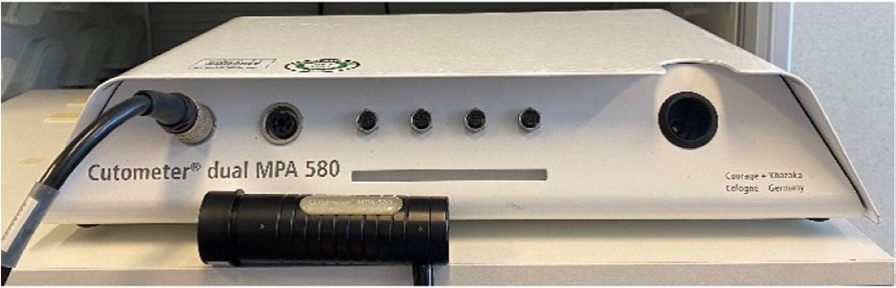
The Cutometer measures scar mechanics retraction, elasticity, viscoelasticity, and maximum extension by inducing negative pressure on scar tissue (Cutometer Skin Elasticity Meter Dual MPA 580 ® Courage and Khazaka GmbH, Cologne, Germany)

The primary outcome measure is the change in scar pliability as measured by the Cutometer Skin Elasticity Meter Dual MPA 580 ® (Courage and Khazaka GmbH, Cologne, Germany) pre- and 12 months post-tSVF-enriched fat grafting (Figs. 2 and 6). The Cutometer® is a validated, reliable instrument which measures viscoelasticity of the skin by analyzing its maximal extension after inducing negative pressure ([33–37], https://www.trialregister.nl/trial/8461).

### Secondary outcome measures

All scar outcome measurements will be performed by two trained observers who work independently of each other to prevent confirmation bias.

#### Scar mechanics

Scar, retraction, elasticity, viscoelasticity, and maximum extension will be measured with the Cutometer Skin Elasticity Meter Dual MPA 580 ® (Courage and Khazaka GmbH, Cologne, Germany) pre-tSVF-enriched fat grafting and 3 and 12 months post-tSVF-enriched grafting (Figs. 2 and 6) [30]. Scar pliability will also be measured after 3 months post-tSVF-enriched fat grafting.

Healthy skin measurements will be conducted as described pre-, 3, and 12 months post-tSVF grafting on the contralateral side of the treated scar. Healthy skin measurements will serve as a reference (i.e., the optimum skin mechanics within that person).

#### Scar color and pigmentation

**Fig. 7:**
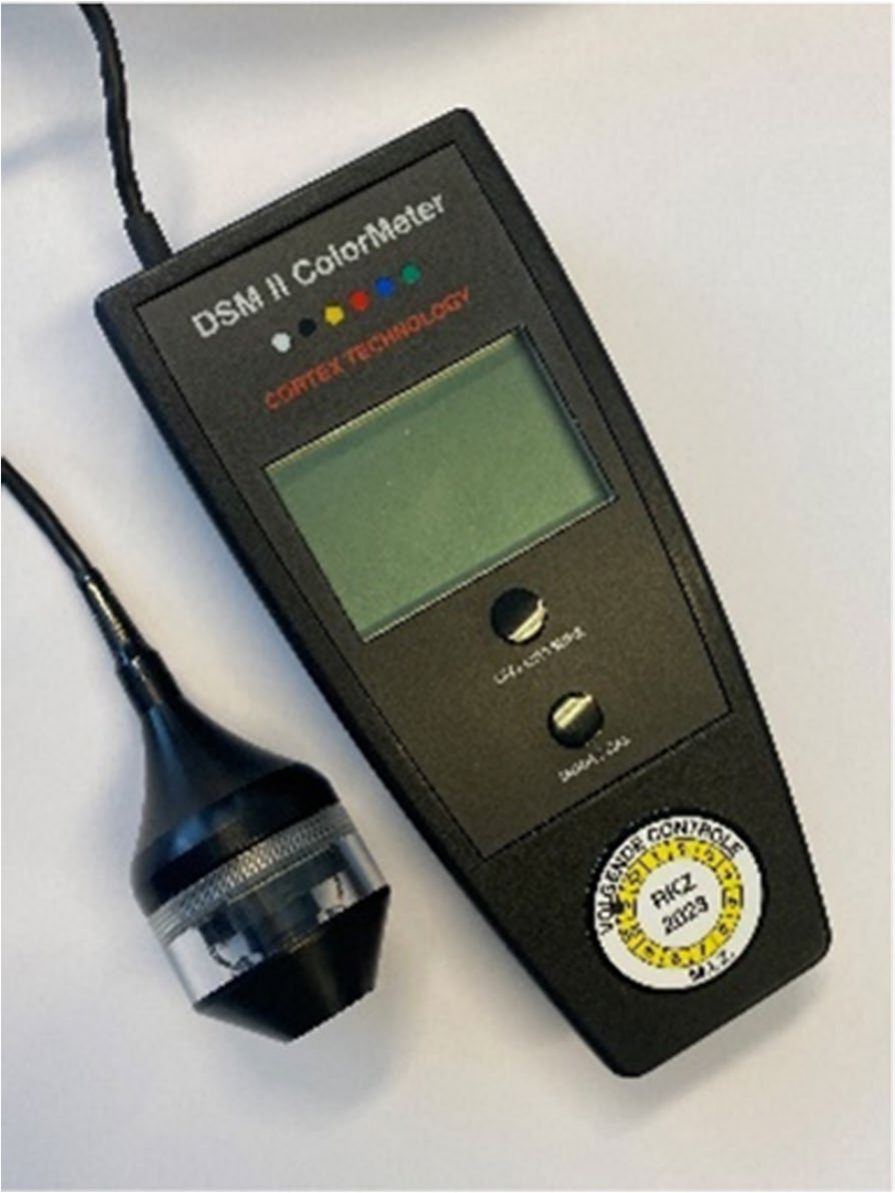
The DSM II Colormeter measures scar color and pigmentation by calculating an erythema and melanin index (Cortex Technology, Hadsund, Denmark)

Scar color and pigmentation will be measured with the DSM II Colormeter ® (Cortex Technology, Hadsund, Denmark), a validated and reliable tool that calculates an erythema and melanin index as a proxy for color and pigmentation (Figs. 2 and 7). Measurements of the selected scar and adjacent normal skin will be performed pre-, 3, and 12 months post-tSVF-enriched grafting [30].

#### Scar quality

**Fig. 8:**
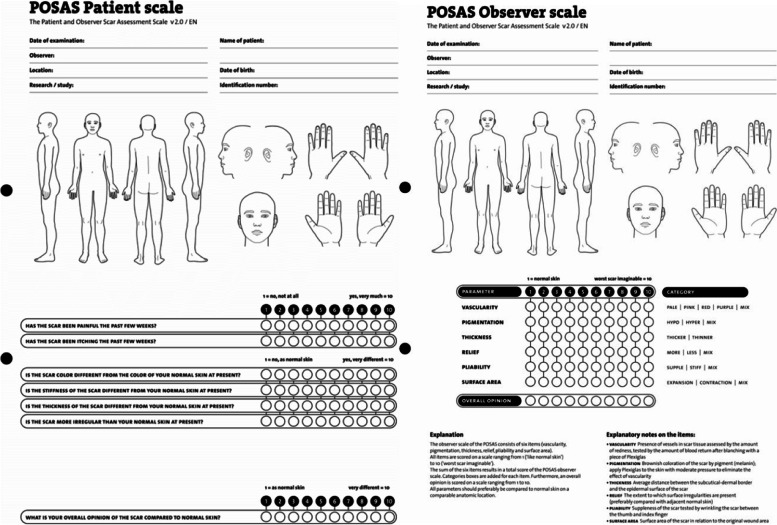
The POSAS Scale 2.0. The patient score combines scar pain, itch, color, stiffness, thickness, and irregularity. The observer scale comprises of the items vascularity, pigmentation, thickness, relief, pliability, and surface area. All items of the patient and observer score combined will form a total POSAS score (the highest score represents the worst scar imaginable). It also consists of an overall opinion of the scar

Scar quality will be assessed by the POSAS version 2.0 ([29], www.posas.org). The POSAS Patient and Observer scales are completed pre-, 3, and 12 months post-tSVF-enriched grafting (Figs. 2 and 8). All items of the POSAS 2.0 will be scored on a 10-point rating scale and added together to obtain a final score. Additionally, item-specific scores and the overall opinion of the scar will be scored (Fig. 8).

#### Standardized photographs

A standardized photograph will be taken from each scar within 24 h before treatment under the same light and camera conditions each time.

#### Histological analyses

For participants in our study, scar biopsies are optional. If patients consent to biopsies, histological features will be assessed by taking a 2-mm biopsy of the selected scar on a fixed position: 1 cm left from point 1 within 24 h before treatment and at 12 months post-tSVF-enriched grafting 1 cm right from point 1 (Fig. 2). The skin sample will be fixed with 2% PFA and paraffin embedded. From each patient, 0.5 ml of tSVF will be 2% paraformaldehyde (PFA) fixed. Fixed samples will be transported to the University and Medical Center Groningen for histological analysis. There, thin sections of paraffin-embedded scar biopsies and tSVF will be deparaffinized and stained with H&E, Picrosirius red (extracellular matrix collagen), CD68 (macrophage infiltration), perilipin A antibodies (presence of residual adipocytes), alpha-smooth muscle actin antibodies (smooth muscle cells and myofibroblasts), and von Willebrand Factor and/or CD31 (endothelial cells). Stained sections will be scanned and subjected to image densitometry for quantification.

#### Baseline patient and scar characteristics

Patients’ baseline characteristics will be extracted from patients’ medical records and consist of the following: age; sex; skin type (Fitzpatrick); scar characteristics, e.g., scar age, cause of the scar; and prior surgery of the selected scar other than fat grafting (exclusion criterion), e.g., split skin grafting.

### Sample size calculation

The primary outcome is the difference in pliability measured by the Cutometer (parameter Ue) between pre- and 12 months post-tSVF-enriched fat grafting. The expected effect size is based on the results of the study by Jaspers et al. on the Coleman fat grafting technique [30]. In this study, scar pliability improved by 22.5%. The mean pliability measure was 0.51. At least this effect is expected to be found in tSVF-enriched fat grafting as well. An additional positive effect of 20% of tSVF-enriched fat grafting (compared to the Coleman technique) is considered a clinically relevant improvement of treatment in this study. Therefore, a sample size calculation was made with an expected effect size of 0.45. With a required minimum of 80% power, a two-tailed test, and *p* < 0.05 considered statistically significant, the required sample size is 41 patients (G*Power 3 version 3.1.9.2). With an anticipated maximum dropout rate of 10% at 12 months, we will need to include 46 patients to compensate for dropout.

### Statistical analysis

Comparisons will be made for all primary and secondary outcome metrics of the selected scar and adjacent normal skin across all follow-up moments, thus pre-, 3, and 12 months post-tSVF-enriched grafting, according to the established data collection protocol [30]. Analyses will be performed using SPSS Statistics, version 27.0 (IBM Corp., Armonk, NY), and Image J (NIH, Behesda, ML) for histological examination of skin biopsies. If data is normally distributed, results will be presented as mean with standard deviation and the primary outcome will be analyses with the paired *T*-test. Furthermore, the repeated measures ANOVA will be performed to detect any overall differences between related means across all time points. If the repeated measures ANOVA is statistically significant, we will run appropriate post hoc tests to highlight exactly where differences occur. If data is not normally distributed, appropriate non-parametric analyses are performed. Data will be analyzed using SPSS, and for histological analysis, GraphPad Prism (version 8.4; GraphPad Software, Inc., La Jolla, USA) will be used. *p*-values < 0.05 will be considered statistically significant.

## Discussion

The aim of this non-randomized intra-patient before-after early phase trial is to assess the efficacy of tSVF-enriched fat grafting on the pliability of adherent scars caused after burn injury or fasciitis necroticans or degloving injury. The potential therapeutic capability of tSVF in adherent scars will be measured by validated objective and subjective scar assessment tools.

This proposed non-randomized study design is part of a larger series of consecutive non-randomized trials with identical study design and standardized algorithm and outcome assessment scheme to allow for identical scar follow-up [30]. The only difference between these studies is the fat grafting technique. These series of studies aim to determine the optimal (fat grafting) treatment for patients with adherent scars. Through the same study design, core outcome set, and patient populations (same inclusion criteria), the efficacy of the various techniques can be systematically compared. Moreover, it will enable historical cohort comparison over time. In this type of cohort series, as compared to classical randomized controlled trials (RCTs), no limiting inclusion criteria are used which provides a better representation of the true patient population to our opinion (real-world evidence). This is in contrast to RCTs in which study populations often only present a small part of the true population due to strict inclusion and exclusion criteria (e.g., exclusion of comorbidities). However, a limiting factor of this study is the lack of a direct comparison of two or more fat grafting techniques under the exact same circumstances as is the case in a RCT. Nonetheless, the real-world data setting that this study provides may result in evidence of the efficacy of tSVF-enriched fat grafting in scar treatment.

In this study, the chosen primary outcome parameter is scar pliability measured by the Cutometer. This scar pliability assessment is a reliable, non-invasive, and painless parameter to assess the therapeutic effects of fat grafting as scar remodeling treatment and the Cutometer is also one of the most researched validated measurement devices to assess tissue pliability and elasticity [39]. Apart from the assessment of objective scar tissue features, inclusion of subjective patient measurements in scar assessment is essential. Subjective scar features are assessed using the validated and world-wide used POSAS questionnaires [26]. Besides the standardized protocol and core outcome set, the power of our trial design is also the proposed combination of clinical and histological analysis of tSVF-treated scar samples. Together, they offer great potential to measure therapeutic efficacy in a real-world setting. This combination will also provide insight in the underlying mechanism of potential regenerative effects of tSVF on scar tissue, which may have direct implications for future research and clinical applications. Moreover, the long follow-up period will reassure that fat, and possibly also tSVF, has initiated regenerative processes and scars have had allowance to reverse to healthier dermal tissue so that this can be objectified with the data outcome set [40].

To ensure equal quality of treatment in the participating centers of this multicenter study and to minimize the effect of confounding factors, several standardization measures were applied. For example, specific durations have been set for procedural steps, e.g., 80 min for normal fat grafting procedures and 20 min for concentrating and injection of SVF-enriched fat grafts. Furthermore, all fat grafting procedures will be carried out according to the Coleman technique and centers will use the same fractionators to manufacture tSVF. Moreover, only plastic surgeons with significant experience with fat grafting procedures will perform the surgeries to ensure equal standard of care. Finally, uniform outcome measures were selected and measurements will be executed by the same trained observers on all follow-up moments (not plastic surgeons to prevent observer or confirmation bias). Patients of the participating Dutch Burn Centers possibly provide an accurate model for burn patients and patients with adherent scars in most high-income countries and the surgical procedure is easily replicable in other like clinical environments where fat grafting can be carried out. This may improve the generalizability of the studies’ conclusions. We believe that our non-randomized multicenter study design is sufficiently powerful to provide such potential evidence that may alter the standard of care. This has precedent, for instance in the non-randomized trial of Jaspers et al., after which fat grafting for adherent scars became insured healthcare in the Netherlands.

In summary, our non-randomized trial will contribute to further optimizing fat grafting treatments of patients with adherent scars and may potentially lead to better and more optimal treatment options for adherent scars than other fat grafting techniques.

## Trial status

This study design is a summary with backgrounds and explanations of the CCMO-approved protocol (version 2.0, 27 August 2020 NL72094.000.20) and complies with the guideline of Good Clinical Practice (GCP). Inclusion opens at December 2021 and is expected to be completed in October 2024.

## Data Availability

Not applicable.

## Abbreviations

ASC: Adipose-derived stromal cell
CCMO: National Dutch Ethical Committee
cSVF: Cellular stromal vascular fraction
FAT: Fractionation of adipose tissue
MSS: Manchester Scar Scale
POSAS: Patient and Observer Scar Assessment Scale
SVF: Stromal vascular fraction
tSVF: Tissue stromal vascular fraction
VSS: Vancouver Scar Scale
